# The mere sight of loved ones does not inhibit psychophysiological defense mechanisms when threatened

**DOI:** 10.1038/s41598-022-06514-y

**Published:** 2022-02-15

**Authors:** Florian Bublatzky, Sabine Schellhaas, Pedro Guerra

**Affiliations:** 1 Central Institute of Mental Health, Medical Faculty Mannheim, Heidelberg University, J5, 68159 Mannheim, Germany; 2 Department of Personality, University of Granada, Granada, Spain

**Keywords:** Emotion, Learning and memory, Classical conditioning, Extinction, Fear conditioning, Human behaviour, Autonomic nervous system, Somatic system

## Abstract

Looking at pictures of loved ones, such as one's romantic partner or good friends, has been shown to alleviate the experience of pain and reduce defensive reactions. However, little is known about such modulatory effects on threat and safety learning and the psychophysiological processes involved. Here, we explored the hypothesis that beloved faces serve as implicit safety cues and attenuate the expression of fear responses and/or accelerate extinction learning in a threatening context. Thirty-two participants viewed pictures of their loved ones (romantic partner, parents, and best friend) as well as of unknown individuals within contextual background colors indicating threat-of-shock or safety. Focusing on the extinction of non-reinforced threat associations (no shocks were given), the experiment was repeated on two more test days while the defensive startle-EMG, SCR, and threat ratings were obtained. Results confirmed pronounced defensive responding to instructed threat-of-shock relative to safety context (e.g., threat-enhanced startle reflex and SCR). Moreover, threat-potentiated startle response slowly declined across test days indicating passive extinction learning in the absence of shocks. Importantly, neither a main effect of face category (loved vs. unknown) nor a significant interaction with threat/safety instructions was observed. Thus, a long-term learning history of beneficial relations (e.g., with supportive parents) did not interfere with verbal threat learning and aversive apprehensions. These findings reflect the effects of worries and apprehensions that persist despite the repeated experience of safety and the pictorial presence of loved ones. How to counter such aversive expectations is key to changing mal-adaptive behaviors (e.g., avoidance or stockpiling), biased risk perceptions, and stereotypes.

## Introduction

The mere sight of significant others—such as pictures of the own romantic partner—has been shown to mitigate the experience of pain and to reduce defensive reflex activity^1,2^. However, very little is known about the modulatory effects of viewing loved ones on the acquisition and extinction of socially transmitted aversive apprehensions. Recent research demonstrated that the mere verbal announcement of potentially threatening events is sufficient to provoke a pattern of pronounced neuronal, somatic and autonomic defensive responses relevant to understanding worry and anticipatory anxiety. Specifically, attention is captured by instructed threat cues and leads to selective allocation of processing resources within a so-called fear network (e.g., ACC, amygdala)^3–5^. This presumably sets the stage for physiological response priming and behavioral actions to cope with anticipated events. For instance, viewing instructed threat relative to safety cues, consistently potentiates the startle reflex and enhances skin conductance responses (SCR)^6–9^. Intriguingly, as a feature of social learning, these bio-behavioral consequences may persist irrespective of whether the anticipated event occurs or not (e.g., across trials, experimental sessions, and even repeated test days)^7,10^. Unlike experiential learning (e.g., Pavlovian conditioning), the acquisition of threat/safety associations in instructional learning does not depend on the personal experience of aversive events, and conversely, the mere absence of aversive events is not a necessary condition for extinction learning (e.g., because the ambiguity of threat expectations persists)^10,11^.

Similar to language, the human face represents a key aspect for social communication. Faces inform about the person’s identity, emotional states, and intentions of people, which are essential cues that help discriminate friendly and hostile social situations. However, to what extent motivational systems are modulated by (emotional) facial information critically depends on the perceived relevance to the observer^12–16^. Recent research started using personalized stimulus materials with particular high implicit self-relevance—pictures displaying significant others such as one’s own romantic partner—within laboratory experimental settings. Similar to the physical presence of loved ones, which has long been associated with physical and psychological well-being^17,18^, the mere pictorial sight of significant others may convey safety. Such safety cues are assumed to simultaneously activate an appetitive reward system and inhibit defense reactions^1,19^. For instance, viewing pictures of the romantic partner has been shown to reduce self-reported pain, diminish pain-related neural activity (e.g., dorsal ACC, anterior insula), and increase activation of areas related to safety-signal and reward processing (e.g., vmPFC)^1,20^.

Even devoid of any emotional expression, loved familiar faces are highly pleasant stimuli. Viewing your loved ones provokes a pattern of psychophysiological changes, which is distinctive of positive emotions and not attributable to familiarity or undifferentiated emotional arousal alone (e.g., enhanced zygomaticus muscle activity, SCRs)^2,21,22^. Moreover, pictures of significant others have been shown to effectively inhibit defensive reflex circuits, leading to reduced startle reflex activity compared to both neutral (unknown) and unpleasant faces^2^. Over and above, some studies suggested that pictures of supportive others might act as evolutionary prepared safety signals, which impede fear conditioning^23,24^. Thus, pictures of loved people may signal safety leading to certain beneficial effects during times of distress. It is unknown, however, whether these findings extend to the inhibitory capacity of loved ones during social threat learning^25^.

Based on previous research on the high stability of verbally instructed threat contingencies over time^7,10^, the present study examined whether loved familiar face pictures may serve as implicit safety cues and attenuate the expression of fear responses and/or accelerate extinction learning during a threatening context (cf.^24^). To follow up on several day extinction processes in social threat and safety learning, experimental sessions were repeated on two more test days^10^. We expected that viewing loved ones would inhibit defensive reflexes, and provoke enhanced autonomic arousal in comparison to unknown faces (i.e., reduced startle reflex and elevated SCRs)^2^. Regarding contextual threat, we predicted pronounced physiological defense activation relative to the safety condition (i.e., threat-potentiated startle reflex and enhanced SCR)^6,7^. These defensive responses were expected to slowly subside across test days, however, with different timing for dependent variables (i.e., most stable threat effects for verbal report, than SCR, and least stable for startle reflex)^10^. Integrating both experimental paradigms—picture viewing and threat-of-shock—we further hypothesized that the expression and extinction of instructed threat responses would vary as a function of face category (unknown vs. loved). As pictures of significant others reduce both subjective and neural responses during the experience of pain^1^, and have been suggested as prepared safety cues^23,24^, a mitigating impact is expected on aversive anticipations. Specifically, a reduction of defensive responding was expected when viewing loved relative to unknown faces (i.e., inhibited startle reflex and threat ratings), especially during threat conditions.

## Methods

### Participants

The sample size was chosen following a previous study that showed stable threat effects over three test days (^10^see Study 2). In addition, estimates using G*Power^26^ suggest that about *N* = 34 was required to detect relevant effects at a medium effect size (f = 0.25), power (1 − β = 0.8), and assumed correlations (*r* = 0.5) across repeated measures in a within-subject ANOVA design. Thirty-three healthy participants (9 males) between the age of 19 and 33 (*M* = 23.97; *SD* = 3.14) were recruited from the population of Mannheim (Germany) and University of Mannheim studentship. One participant did not show up for the second and third test day, thus the final sample was N = 32. EMG data of one participant was overly noisy (i.e., 43% missing trials) and another participant did not show SCRs; these participants were excluded from the respective startle and SCR analyses (*N* = 31).

As inclusion criteria, participants were prescreened via telephone for having a lasting partnership (for at least 6 months; *M* = 39.64, *SD* = 26.93) and a good relationship quality to their ‘loved ones’ (i.e., reporting quality of at least 70%, on a scale of 0–100; cf.^2^). Accordingly, relationship quality was high with the partner (*M* = 90.91, *SD* = 6.69), parents (*M* = 89.24, *SD* = 8.92), and best friend (*M* = 90.45, *SD* = 9.46). None of the participants indicated that their relationship quality had changed (e.g., due to an argument) within the 3 days of testing. Additional questionnaires regarding state-trait anxiety, social anxiety, depression, and relationship quality were completed before the first testing day online via SoSci-Survey software (www.soscisurvey.de).

Exclusion criteria were previous participation in a threat-of-shock study, acute or chronic psychiatric or medical conditions, hearing or vision problems, taking psychotropic drugs, pregnancy, and having a medical advice to avoid stressful situations. All participants, and their attachment figures, were informed about the study protocol and provided written informed consent. Thirty-five Euro expense allowance was paid for participation. The ethics committee of the Medical Faculty Mannheim, Heidelberg University (Germany) approved the experimental protocol, which complies with the APA ethical standards and the Declaration of Helsinki.

### Materials and design

**Figure 1:**
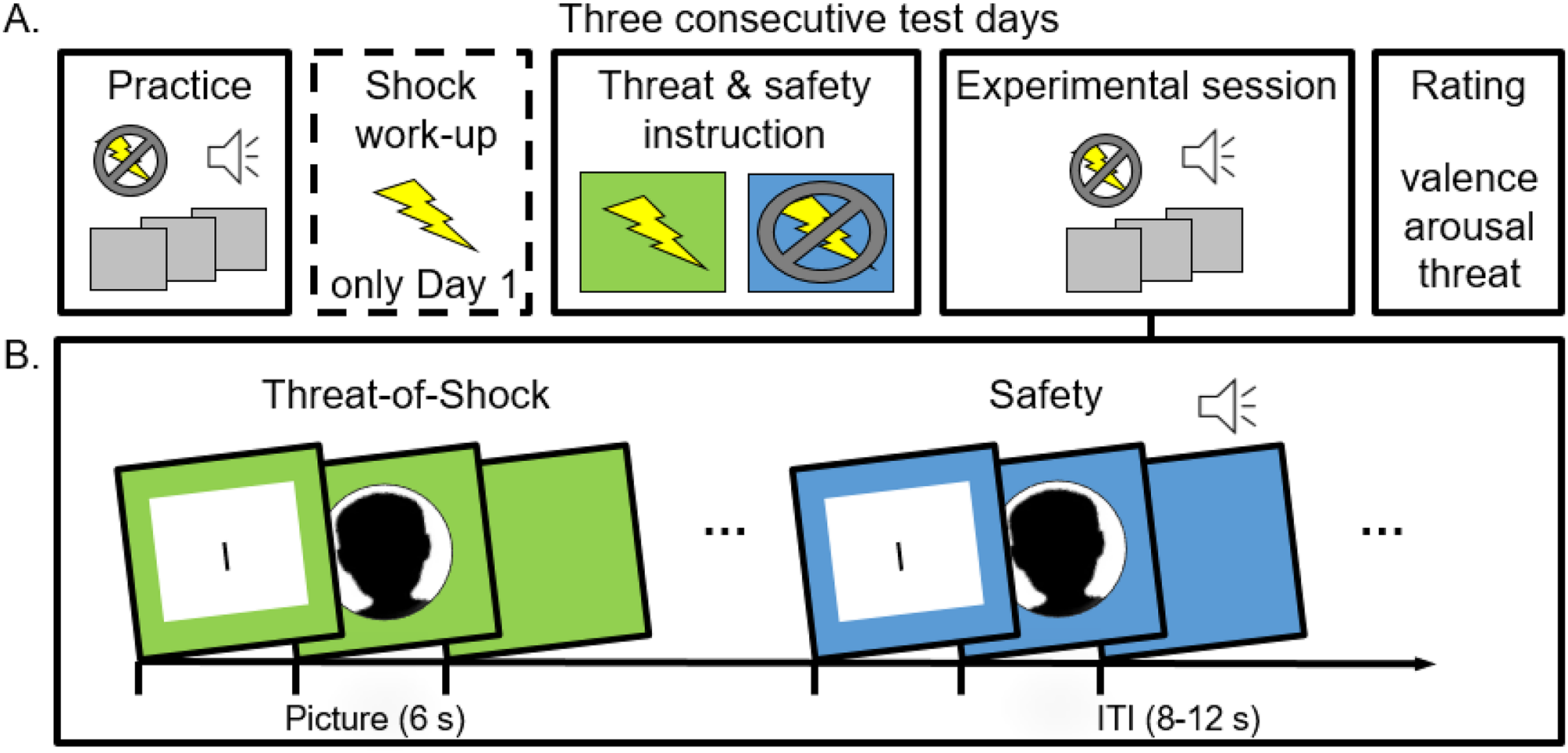
Schematic illustration of the experimental procedure. (**A**) Following initial practice trials, a shock work-up procedure was carried out to ensure credibility of the threat-of-shock instructions. Then the experimental session started and afterwards context conditions as well as picture materials were rated. The procedure was completed on three consecutive test days (except for the shock work-up, which was performed only on Day 1). (**B**) Each experimental session consisted of alternating blocks of instructed threat or safety as indicated by background colors. In each block all faces were presented once (i.e., 4 loved people and 4 unknown faces). Blocks were preceded by instruction slides (I) as a brief reminder.

Personalized picture materials were used for each participant. To this end, the romantic partner, mother, father, and best friend of each participant provided personal profile photographs with neutral expressions. Before the experiment, these personalized face pictures were resized (866 × 866 pixels), cropped with an elliptic mask around the face, transformed to grey-scales, and adjusted for background and luminance (see Fig. 1). To keep the ratio of female to male face pictures constant, only participants with opposite-sex romantic partners and same-sex best friends were included. Importantly, personalized face pictures, provided by one participant (i.e., the loved face category) were used as unknown control pictures for exactly one other participant (i.e., participant N's unknown control pictures were participant N − 1's loved face pictures). This tandem-use of the pictures ensured comparability of stimulus materials across participants.

Participants completed the experimental sessions on three consecutive test days. Each session consisted of eight alternating threat and safety blocks, lasting for approximately 2 min each (cf.^10^). Threat and safety condition were indicated by contextual background colors (green or blue; 786 × 1024 pixels) surrounding the pictures. Participants were verbally instructed that they might receive up to three unpleasant but non-painful electric shocks when one specific color was presented (e.g., green signaling threat), but not when the other color was presented (e.g., blue signaling safety). Color assignment to condition and order of first shock/safety condition were balanced across participants. A written reminder of condition (i.e. “shock possible” or “no shock”) preceded each block for 6 s.

Each block depicted eight picture trials displaying all face pictures exactly once for 6 s, followed by a varying inter-trial interval (ITI; 8, 10 or 12 s). In half of all picture trials (i.e., 32), an auditory startle probe (white noise 105 dB, 50 ms) was presented at 4, 4.5, or 5 s after picture onset, equally distributed across picture categories and blocks. In addition, eight probes were delivered during ITI. Mean time lag between startle probes was 25.6 s.

Pictures were presented on a 22-inch monitor located approximately 70 cm in front of the participants. Startle probes were delivered binaurally via headphones (AKG K44), and electrical stimuli (maximum 10 mA, 100 ms) were administered to the non-dominant inner forearm using a Digitimer DS7A stimulator (Digitimer Ltd., Welwyn Garden City, UK). Stimulus control was accomplished by using Presentation software (Neurobehavioral Systems, Berkeley, CA).

### Procedure

Participants came to the laboratory on three consecutive days approximately at the same time of day. After sensors were attached, initial practice trials consisting of the presentation of eight habituation startle probes (not analyzed) served to familiarize participants with the experimental procedure. After the electrical stimulator electrode was attached, a brief shock work-up procedure was carried out on test Day 1 only (cf.^10,27^). Participants received up to 10 electrical shocks with increasing intensity until stimulation was reported as ‘maximally unpleasant but not yet painful’. Building upon this, participants were told that the intensity of the electric stimulation during the experiment would be equal to the most unpleasant test shock. Then, instructions regarding the association of frame colors and threat or safety condition were given and the experiment started with the first experimental block. On each test day, participants’ task was to passively view all pictures presented on the screen. Halfway through the experimental sessions, a brief break was included and at the end of each session, participants rated context conditions and picture materials regarding valence, arousal, and perceived threat. Except for the shock work up, the same procedure was repeated on Day 2 and 3 and no shocks were given across all test days (cf.^10^). The pairings of threat/safety-colors were always constant within one participant. A final debriefing interview was completed at the end of the last test day.

### Data recording and reduction

Psychophysiological measures were continuously recorded with a Biopac amplifier and AcqKnowledge software (BIOPAC Systems; Goleta, CA). Startle amplitudes were derived from the electromyogram of the orbicularis muscle by means of two Ag/AgCl electrodes attached below the right eyelid^28^. The raw signal was recorded at a 1000 Hz sampling rate, and frequencies below 28 Hz and above 500 Hz were filtered out with a band-pass filter (24 dB/octave roll-off). Finally, a 50 Hz Notch filter was applied to remove potential contamination coming from the power-line. Data were subsequently rectified and smoothed with a moving average procedure (50 ms) in VisionAnalyzer 2.0 (BrainProducts, Munich, Germany). Startle responses were scored with an automated procedure as maximum peak in the 21–150 ms time window following each startle probe, and peak amplitude calculated relative to a mean baseline period (50 ms preceding startle response time window; cf.^10^).

As an index of phasic autonomic activation, SCRs to startle probes were recorded with Ag/AgCl electrodes (constant voltage of 0.5 V; 20 Hz sampling rate) placed at the hypothenar eminence of the non-dominant hand. Noise was attenuated using a Butterworth Zero Phase 2 Hz low- and a 0.05 Hz high-pass filter. SCRs to startle probe onset were calculated as the maximum increase in skin conductance in the interval of 1–4.5 s (relative to a 2 s pre-stimulus period). A minimum threshold of 0.02 µS was used for zero-response detection, and range and distribution correction were applied within each participant [square root (response/maximum response)].

Picture materials and contextual background colors indicating threat-of-shock or safety were rated. Self-reported valence and arousal were obtained using the Self-Assessment Manikin (SAM)^29^, a non-verbal pictorial assessment technique ranging from *unpleasant* to *pleasant* and *calm* to *highly arousing* (1–9). Perceived threat was measured using a visual analog scale ranging from *not at all* to *highly threatening* (0–10) at the end of each session.

### Data analysis

Data and syntax can be retrieved here: https://osf.io/5q9an/?view_only=ff069eab45f142bbaca9017fb1fcecc7.

The same statistical design was used for the analyses of startle reflex and skin conductance responses (to startle probes), as well as the self-reported threat, valence, and arousal ratings for picture materials and contextual background colors using SPSS (Version 27). To this end, separate repeated measures ANOVAs were conducted including the within factors Context (threat vs. safety), Face Category (loved vs. unknown), and Day (Day 1 vs. Day 2 vs. Day 3). Separate post hoc *t*-tests were conducted to follow up on significant interactions.

We further conducted Bayesian analyses to gain more information about non-significant effects of our key hypothesis (i.e., estimating the probability of the null relative to alternative hypotheses)^30^. To this end we focused on the of-interest interactions Context × Category × Day. Bayes factors (BF) were estimated for all relevant models (Context, Category, Context × Category, Context × Day, and so on; see Table 1) using Monte-Carlo sampling 10,000 iterations and default prior scaling factors (for fixed effects = 0.5, random effects = 1, r covariates = 0.354)^31^ using the R based software package JASP^32^. We report BF inclusion scores (BF_incl_) that inform about how much the inclusion of a factor (e.g., Context, averaged across all models that include Context as a factor) is supported by the data, compared to all other models (including the null-model). A value of 1 indicates that both null- and alternative hypotheses are equally probable with the data at hand, while values below (or above) 1 suggest that the data are more (or less) likely under the null-relative to the alternative hypothesis. For example, a BF < 0.333 indicates that the data is at least three times more likely under the null- compared to the alternative hypotheses (and vice versa for a BF > 3).

**Table 1:** Bayes factors (BF_incl_) of the selected models compared to all models without this factors for the different dependent measures.

Model BFInclusion:	Context rating			Picture rating			Physiology	
	Threat	Valence	Arousal	Threat	Valence	Arousal	Startle	SCR
Context	3.160 × 10^14^	3.16 × 10^14^	3.125 × 10^11^	∞	1.901 × 10^7^	2.145 × 10^14^	2.137 × 10^7^	27.927
Face Category				6.143 × 10^8^	5.106 × 10^13^	93,125.272	0.07	0.066
Day	1.544 × 10^6^	0.081	511,112.697	2.087 × 10^8^	0.064	49,555.602	1.476 × 10^13^	474.082
Context × Face Category				0.337	0.381	0.432	0.051	0.049
Context × Day	16.523	0.074	13.724	17.292	0.03	1.373	5.616	3.872
Face Category × Day				0.292	0.106	0.354	0.018	0.067
Context × Face Category × Day				0.026	0.001	0.024	0.002	0.006

Greenhouse–Geisser corrections were applied where necessary, and the partial eta square (η_p_^2^) is reported as a measure of effect size. To control for type 1 error, Bonferroni correction was applied for post hoc t tests.

## Results

### Self-report data

**Figure 2:**
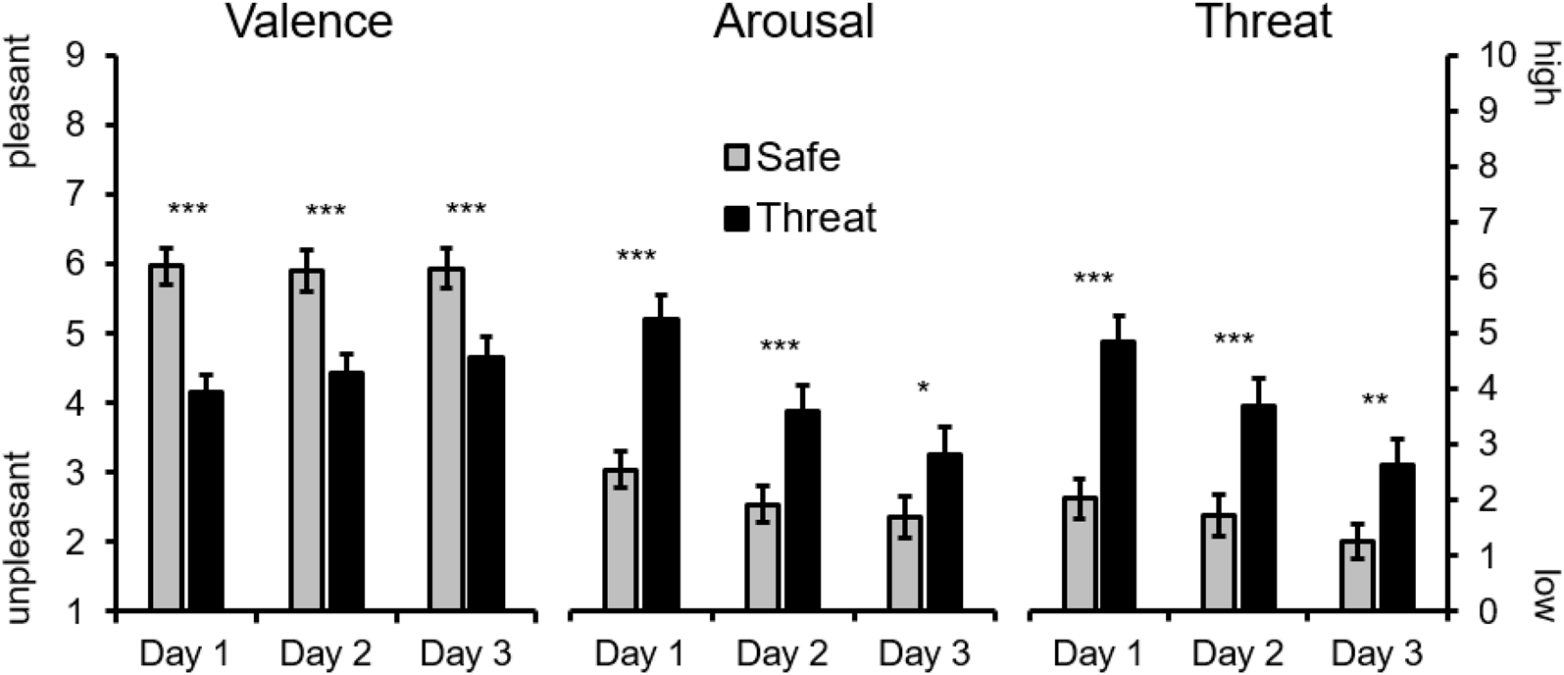
Self-reported valence, arousal and threat ratings show highly persistent effects of instructed threat compared to safety context across three test days (M and SEM, ***p < 0.001, **p < 0.01, *p < 0.05).

Similar to previous research, the contextual background color serving as signal for threat-of-shock was rated as more unpleasant, arousing, and threatening compared to the safety background, *Fs*(1,31) = 35.98, 38.21, and 41.40, *ps* < 0.001, ƞ_p_^2^ = 0.54, 0.56, and 0.57 (see Fig. 2). For valence ratings neither Day, *F*(2,62) = 0.88, *p* = 0.40, ƞ_p_^2^ = 0.03, nor the interaction Day × Context reached significance, *F*(2,62) = 2.57, *p* = 0.10, ƞ_p_^2^ = 0.08, BF_incl_ = 0.074, with the null hypothesis being 13.51 times more likely than the alternative interaction hypothesis. However, arousal and threat ratings were reduced across Day, *Fs*(2,60) = 16.52 and 27.01, *p* < 0.001, ƞ_p_^2^ = 0.36 and 0.47, and also revealed interaction effects Context × Day, *Fs*(2,60) = 11.59 and 13.88, *p* < 0.001, ƞ_p_^2^ = 0.28 and 0.31, BF_incl_ = 13.72 and 16.52, which indicate decreasing context effects across testing days. Nevertheless, follow-up test for each test day separately show significant instructed threat effects for arousal, *ps* < 0.05, and threat ratings, *ps* < 0.01.

Face pictures presented within a threat context were perceived as more unpleasant, arousing, and threatening compared to pictures presented with the safety color as backdrop, *Fs*(1,31) = 18.63, 25.20, and 30.31, *ps* < 0.001, ƞ_p_^2^ = 0.38, 0.47, and 0.49. Moreover, compared to loved familiar faces, pictures of unknown people were rated as more unpleasant, arousing, and threatening, *Fs*(1,31) = 49.27, 18.82, and 28.02, *ps* < 0.001, ƞ_p_^2^ = 0.61, 0.39, and 0.48. For valence ratings, instruction effects did neither vary across test days, Context × Day *F*(2,62) = 1.76, *p* = 0.19, ƞ_p_^2^ = 0.05, nor for Context × Face Category *F*(1,31) = 1.27, *p* = 0.27, ƞ_p_^2^ = 0.04. In contrast, arousal and threat ratings were reduced across test days, Context × Day *Fs*(2,58) = 4.66 and 11.67, *ps* < 0.05 and 0.001, ƞ_p_^2^ = 0.14 and 0.27, however, follow-up test show significant context effects for each test day separately, for arousal and threat ratings all *ps* < 0.01. No Context × Face Category interactions emerged for arousal or threat ratings, *Fs*(1,29) = 2.41 and < 0.01, *ps* = 0.13 and 1.0, ƞ_p_^2^ = 0.08 and < 0.01; for each test day separately, unknown faces were rated as more arousing and threatening compared to loved familiar faces, *ps* < 0.001. No three-way interaction Context × Face Category × Day were observed for none of the rating dimensions, *Fs*(2,58) < 2.56, *ps* > 0.09, ƞ_p_^2^ < 0.08, BFs_incl_ < 0.026, indicating that the null hypotheses are at least 38.46 times more likely than the alternative interaction hypotheses. Exploratory analyses focusing on within-category comparisons (e.g., girl/boy-friend vs. mother/father) can be found in the supplementary materials.

### Startle reflex

**Figure 3:**
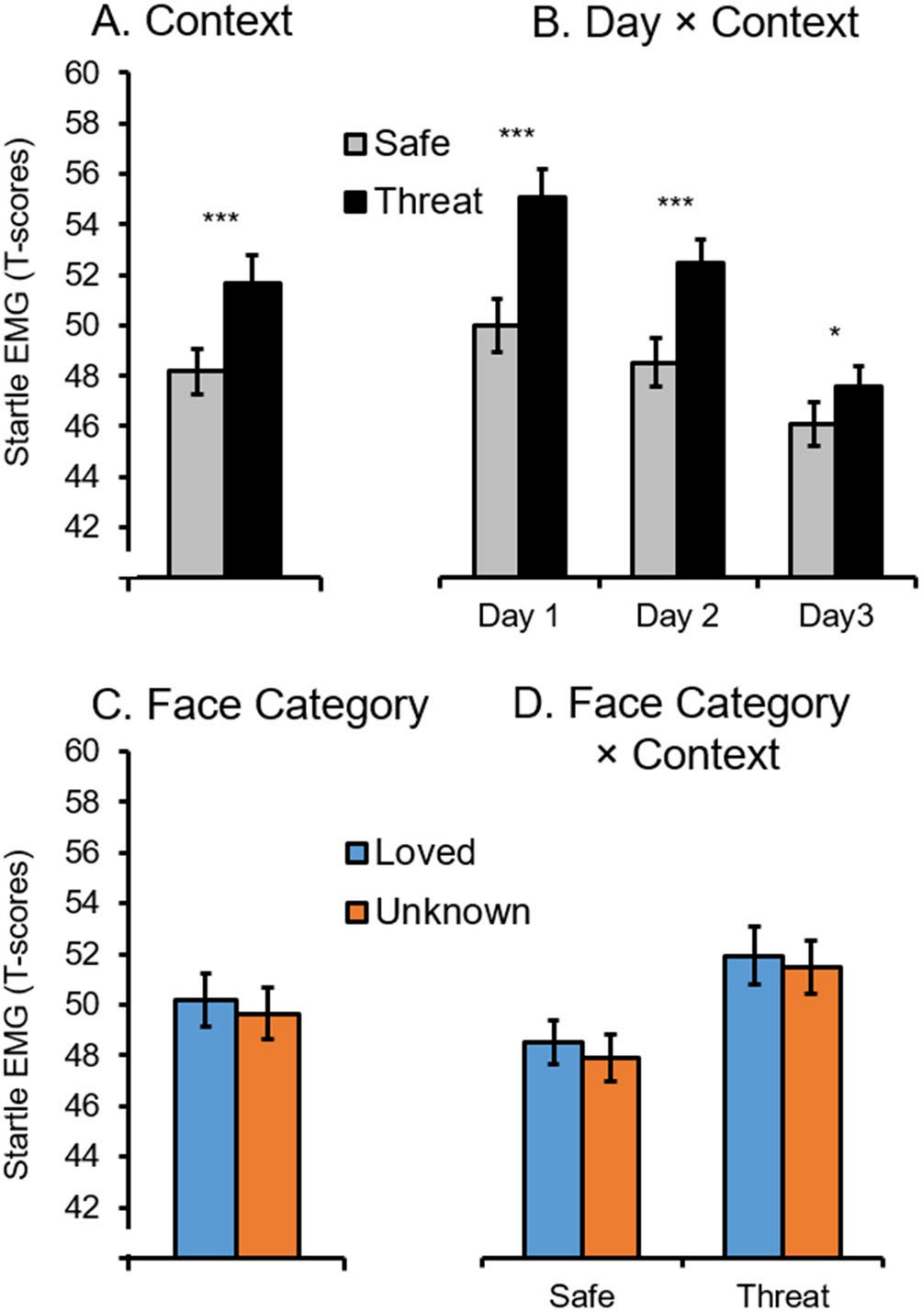
Mean startle reflex as a function of (**A**) instructed context condition, (**B**) context across test days, (**C**) face category, and (**D**) interaction of face category by context condition (M and SEM, ***p < 0.001, *p < 0.05).

Contextual threat signals potentiated the defensive startle reflex relative to safety condition, Context *F*(1,31) = 28.57, *p* < 0.001, ƞ_p_^2^ = 0.48 (see Fig. 3). Moreover, reflex amplitudes decreased across test Days, *F*(2,62) = 9.7, *p* < 0.001, ƞ_p_^2^ = 0.24, and revealed a significant interaction of Context × Day, *F*(2,62) = 10.01, *p* < 0.001, ƞ_p_^2^ = 0.24. Follow-up tests show threat-potentiated startle reflex for Day 1 and 2, *ps* < 0.001, and significant but less pronounced for Day 3, *p* < 0.05.

In contrast to our hypotheses, the startle reflex was not modulated by Face Category, *F*(1,31) = 2.92, *p* = 0.10, ƞ_p_^2^ = 0.09, BF_incl_ = 0.07, nor varied as a joint function of Context × Face Category *F*(1,31) = 0.01, *p* = 0.92, ƞ_p_^2^ < 0.01, or Context × Face Category × Day, *F*(2,62) = 0.02, *p* = 0.97, ƞ_p_^2^ < 0.01, BFs_incl_ = 0.051 and 0.002, with the respective null hypotheses 19.61 and 500 times more likely than the alternative interaction hypotheses.

### Skin conductance responses

**Figure 4:**
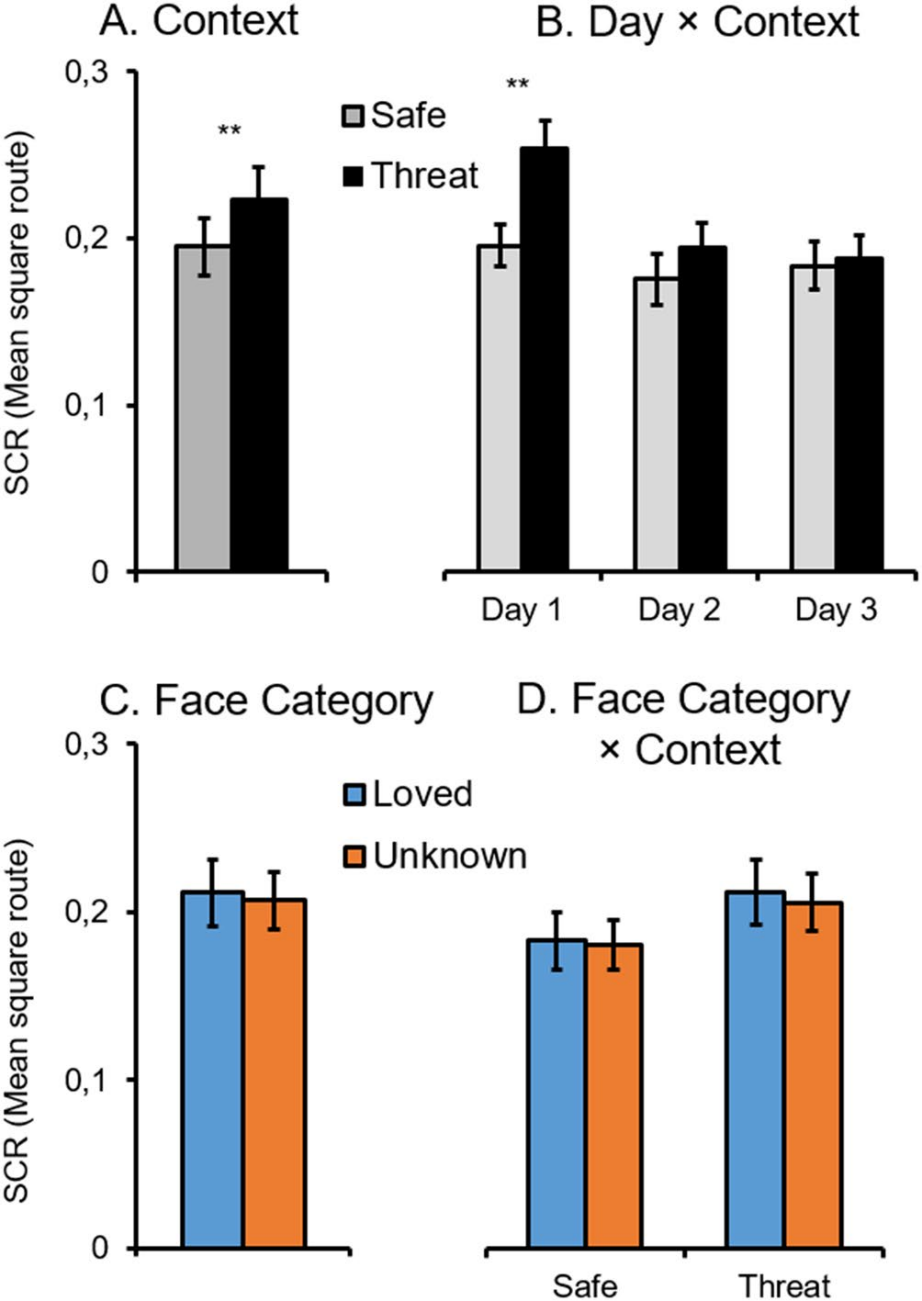
Mean skin conductance responses as a function of (**A**) instructed context condition, (**B**) context across test days, (**C**) face category, and (**D**) interaction of face category by context condition (M and SEM, **p < 0.01).

Threat-enhanced SCRs to startle probes were observed when viewing pictures presented during threat relative to safety, Context *F*(1,30) = 10.37, *p* < 0.01, ƞ_p_^2^ = 0.26 (see Fig. 4). Across test days, this context effect diminished, Context × Day, *F*(2,60) = 4.53, *p* < 0.05, ƞ_p_^2^ = 0.13, showing significant threat effect on Day 1, *p* < 0.001, but not on Day 2 and 3, *ps* = 0.18 and 0.64. SCRs did not differentiate between loved and unknown faces, Face Category *F*(1,30) = 0.30, *p* = 0.59, ƞ_p_^2^ = 0.01. Neither an interaction Context × Face Category was significant, *F*(1,30) = 0.05, *p* = 0.82, ƞ_p_^2^ < 0.01, BF_incl_ = 0.049, nor the overall interaction Context × Face Category × Day, *F*(2,60) = 0.33, *p* = 0.71, ƞ_p_^2^ = 0.01, BF_incl_ = 0.006, with the null hypotheses 20.41 and 166.67 times more likely than the alternative hypotheses.

## Discussion

The present study tested the hypotheses that viewing loved familiar face pictures during times of aversive apprehension would reduce the impact of contextual threat signals and/or facilitate the reduction of threat effects across repeated test days. No support for these hypotheses was observed for any of the dependent variables. Similar to previous work, pronounced activation of the autonomic and somatic response systems emerged within a context of threat-of-shock relative to safety (i.e., enhanced skin conductance responses and potentiated startle reflex)^7,8,10,33^. These threat effects declined across test days but remained significant through the end of test day 3 for the startle reflex as well as threat and arousal ratings. Moreover, the impact of contextual threat was not modulated by the face category (i.e., loved vs. unknown) that was presented concurrently in the foreground. Specifically, viewing loved ones did neither modulate the threat-potentiated defensive startle reflex, enhanced skin conductance responses, nor it had an impact on the affective ratings of the threat and safety context conditions. Compared to unknown people, pictures of loved ones were perceived as more pleasant, less threatening and arousing, but these positive aspects did not affect the functioning of defensive physiological response systems during threat. Taken together, the effects of socially learned aversive anticipations were not reduced by viewing facial images of loved ones, such as one's romantic partner.

The knowledge that a particular person or situation is potentially dangerous triggers caution, aversive anticipation, and prepares avoidance or defense behavior^6,8,25,34,35^. Threat-related psychophysiological responding here involves preparatory activation of the somatic and autonomic nervous systems, and is adaptive in that potential harm to the organism can be avoided or reduced. However, if the expected aversive event does not occur (e.g., persistent absence of expected shocks), this does not necessarily mean that the situation is completely safe. Accordingly, defense systems remain prepared, and this is exactly what we observed. Despite the lack of shock occurrence across three test days, threat-potentiated startle reflex and more aversive self-report were found for threat relative to safety contexts until the end of the experiment. This finding replicates previous studies that show persistent effects of instructional threat learning^7,10^ and contrast with rather quick extinction learning in studies that use shock reinforcement to acquire threat associations (e.g., Pavlovian conditioning). Thus, extinction of the pure cognitive representation of instructed threats (i.e., 0% shock reinforcement) seems to require more than the mere experience of safety (i.e., the continued absence of shocks).

Here, we examined whether viewing images of loved ones may reduce the impact of co-occurring threat signals. In contrast, our results show that pictures of loved ones neither modulated contextual threat effects (i.e., threat-potentiated startle reflex regardless of face category) nor did the physiological responding differ between pictures of loved or unknown faces. These results, based on well-powered classical null hypothesis significance tests (N = 32 * 3 test sessions = data from 96 measurements), are supported by Bayesian analyses showing that for startle reflex and skin conductance, the null hypotheses (i.e., no interaction between face category and context) are 19.61 and 20.41 times more likely than the alternative interaction hypotheses. Moreover, this non-interactive pattern between face category and threat/safety learning goes in line with several recent studies. For instance, using personalized stimulus materials as explicitly instructed threat cues did not show an inhibition of somatic and autonomic responding towards loved faces (i.e., pictures of the spouse)^25^, and this was further replicated with pictures displaying smiling or angry-looking loved ones (Guerra et al., in prep.). Thus, accumulating evidence suggests that (at least in the present experimental approach) viewing loved faces does not lead to inhibition of defensive reflex activity or of the autonomic nervous system.

While some studies suggested a resistance of supportive others against becoming threatening (i.e., showing no differential conditioning)^23,24^, the overall pattern here does not support the notion that attachment figures are per se prepared safety cues. Indeed, this would be in line with the notion that the human face (compared to pictures of snakes or spiders)^36,37^ appears to be a less reliable source of implicit or inherited threat or safety information. For example, social acquisition of threat and safety associations appears to function equally well when linked to facial expressions (e.g., happy or angry faces as threat cues)^34,38^ and facial identity information, even for beloved familiar faces^25,35^. Thus, psychophysiological responding reflects a defense mechanism that reacts faithfully to the threatening situation (i.e., colored background), while disregarding and possibly overshadowing prominent foreground information (i.e., pictures of loved ones) that is non-diagnostic with respect to the anticipated danger (i.e., shocks).

Nonetheless, our findings seem surprising given the many positive effects of caregivers, attachment figures, and significant others on physical health, well-being, and affect regulation^18,20^. For instance, holding hands with your spouse reduces reported unpleasantness of shock anticipation compared to no hand-holding or holding hands with a stranger^39,40^. Similarly, the mere pictorial presence of loved ones (e.g., pictures of the spouse) has been hypothesized to activate a mental representation of that person, resulting in reduced pain^1,41^, and inhibition of defensive reflex activity^2^. With regard to the impact of significant others on learning mechanisms, the evidence is mixed and this may rely on the basic function of learning. Specifically, learning enables an individual to constantly re-align to changes in the external (social) world. Here, a ‘better safe than sorry’ strategy seems to be evolutionary more advantageous (i.e., to avoid future harm) than prioritizing attachment figures as safe by default.

Another question that arises is why viewing loved ones did not inhibit defensive responding during contextual threat^2^. While pictures of loved faces were rated as less unpleasant, arousing and threatening compared to unknown faces, this overall difference did neither modulate ratings or psychophysiological responding toward contextual threat signals, nor did it vary across test days. Autonomic and somatic responding triggered by real-world threats (i.e., acoustic startle probes and shock threat) might have been less susceptible to the mental representations of loved ones. Alternatively, the mere pictorial presence of loved ones was not perceived as helpful and/or supportive compared with the real presence of a supportive person. In addition, certain individuals within the category of loved faces may be more helpful and better threat or safety signals (e.g., romantic partner vs. father^19,21^; see also supplements). To test these hypotheses, future research may directly compare in-person, in-video, or in-picture presence of selected individuals. Here, the physical presence or absence (e.g., in case of loss and mourning for an important person) as well as the type of pro-social or helping behavior (e.g., verbal social support or social touch)^42,43^, might be more relevant factors than the person offering support.

One of the strengths of the present study relates to the use of personalized stimulus materials, which has the advantage of engaging the participants' own social system in a laboratory setting. However, this approach also has its limitations and constraints of generality. For instance, our selection of ‘loved ones’ always included photos of parents, romantic partner, and a best friend, provided that high relationship quality was reported^19,21,22^. While high relationship quality does not necessarily imply perceived high social support (an often used selection criteria)^1^, we could control for differences in familiarity, gender, and age of the used face pictures^19,44^. Moreover, we edited all pictures to depict close-ups, crops with an elliptical mask around the face, and grey-scaled faces with neutral expressions. This was done in order to reduce the influence of different situational and contextual information of the facial images, while preserving identity-related and characteristic facial information (e.g., craggy facial features, hairstyle, and glasses). However, these efforts to standardize face pictures also resulted in a less naturalistic experimental situation^45^, which may have reduced the inhibitory effects of the ‘loved face’ category.

Finally, and with regard to the used verbal learning paradigm, threat and safety contingencies were explicitly instructed and kept constant within participants and across experimental blocks and sessions. However, verbal and written statements were repeated throughout the experiment and thus reinforced shock expectancy while no shocks were actually administered. This constant violation of expectations may play a crucial role in reinforcing attentional, perceptual, and response biases toward threat^46,47^. Here, future research may directly focus on online expectancy ratings as a function of instructional threat and safety learning^48,49^. Moreover, addressing the differential role of active safety learning^50,51^, and distinguishing the role of imminent threat omission and safety learning may be particularly interesting for a better understanding of social learning and its relevance for successful treatment of anxious psychopathology^52–54^.

From a clinical perspective, the present findings may reflect the effects of worries and apprehensions related to anticipatory anxiety, that persist despite the experience of safety and pictorial presence of loved ones. How to counteract such fears and anxieties, that cannot be 100% disproven, is key to changing mal-adaptive behaviors (e.g., avoidance or stockpiling). This is important for providing more effective prevention and intervention programs in clinical settings^53,55^, but also for public service advertising and changing risk perceptions, attitudes, and stereotypes (e.g., through media coverage)^56,57^. Future research needs to address the interindividual antecedences (e.g., threat knowledge and beliefs), resilience factors (e.g., social network), and (sub)clinical fear and anxiety in more detail (see also supplements); here, the inclusion of personalized stimuli and situations in the experimental procedure could be particularly helpful.

In summary, across three repeated test days, instructed threat effects persisted despite the complete absence of aversive shocks. Moreover, no influence was observed for the co-occurring safety cues (i.e., pictures of beloved faces), providing little evidence for implicit downregulation of defensive responses when viewing pictures of beloved familiar people. These data indicate the high persistence of socially acquired threat information over time, especially in the absence of additional safety signals.

## Supplementary Information

Supplementary Information.
