# Supplementary material for: The mere sight of loved ones does not inhibit psychophysiological defense mechanisms when threatened
Source: Sci Rep. 2022 Feb 15;12:2515. doi: 10.1038/s41598-022-06514-y (PMC8847570; doi:10.1038/s41598-022-06514-y)

# Supplementary material to the manuscript:

The mere sight of loved ones does not inhibit psychophysiological defense mechanisms when threatened

Florian Bublatzky ^1, 2^ *, Sabine Schellhaas ^1^, & Pedro Guerra ^2^

^1^ Central Institute of Mental Health, Medical Faculty Mannheim, Heidelberg University, Germany

^2^ University of Granada, Department of Personality, Spain

## Content:

Supplement 1: Picture ratings within-category comparisons

Supplement 2: Summary of covariation effects with questionnaire data

## Supplement 1: Picture ratings within-category comparisons

While the present study does not allow for examining within-category effects of physiological responding (i.e., number of trials per condition and participants precludes meaningful statistics), supplementary analyses of the picture ratings included the exploratory factor Identity (father, mother, partner, and best friend; for details and re-analyses see OSF link to data and syntax, <https://osf.io/5q9an/?view_only=ff069eab45f142bbaca9017fb1fcecc7>). A significant main effect of Identity emerged for valence and threat ratings, *Fs*(3,93) = 3.58 and 3.24, *ps* = .026 and .031, ƞ_p_^2^ = .10, and marginally for arousal, *F*(3,87) = 2.84, *p* = .06, ƞ_p_^2^ = .09. Pairwise comparisons indicate that the parents (especially pictures of the fathers) were rated generally as more unpleasant, arousing and threatening as compared to pictures of the partner and best friend. However, this effect did not vary as a function of Face Category × Identity, *Fs* = .40, .48, and .23, *ps* > .59, ƞ_p_^2^ < .02.

Interestingly, for threat ratings an interaction Context × Identity emerged, *F*(3,93) = 6.32, *p* = .002, ƞ_p_^2^ = .17, indicating that within a threat context, father faces were rated significantly more threatening compared to the faces of partners or best friends, *ps* < .05, who did not differ from each other, *ps* > .19. However, no such interaction was observed for valence or arousal ratings, Context × Identity, *Fs* = 1.53 and 1.3, *ps* > .22, ƞ_p_^2^ < .05. The higher-order interaction Context × Face Category × Identity was not significant for valence, arousal, or threat ratings, *Fs* = .54, 1.03, and 1.29, *p* = .63, .36, and .28, ƞ_p_^2^ = .02, .03, and .04.

The only other significant interaction including identity was observed for threat ratings, Day × Identity, *F*(6,186) = 2.68, *p* = .037, ƞ_p_^2^ = .08. Pairwise comparisons for the first test day show that fathers were perceived as more threatening compared to partners and best friends, *ps* = .005 and .014, no difference emerged between the other identities, or for the other test days, *ps* > .154.

## Supplement 2: Summary of covariation effects with questionnaire data

Exploratory analyses were conducted to test for covariations between psychophysiological measures and dimensional measures of the relationships (relationship quality and duration) and questionnaire data (STAI-S, STAI-T, SPIN, SIAS, FNEK, BDI). The table below summarizes several significant covariation effects. For further details and re-analyses see OSF link to data and syntax <https://osf.io/5q9an/?view_only=ff069eab45f142bbaca9017fb1fcecc7>


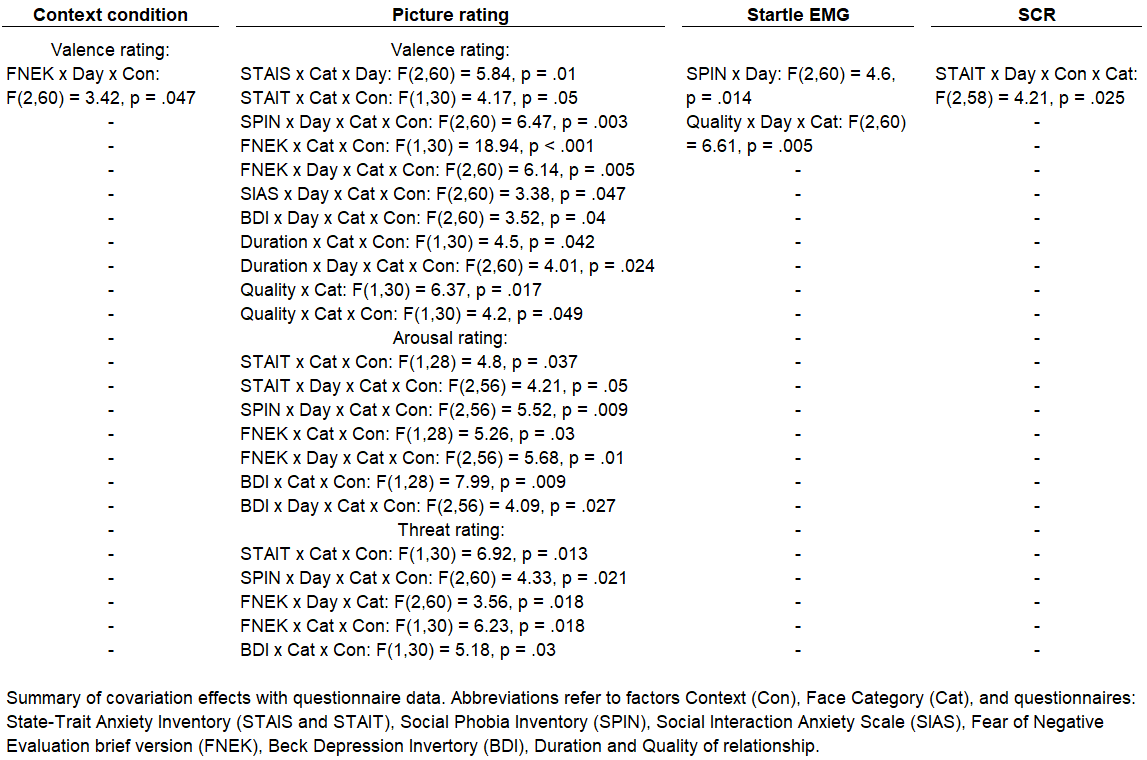

Supplement: Supplementary file 1 — Supplementary Information. [file 41598_2022_6514_MOESM1_ESM.docx]
